# Risk of solid cancers among users of clozapine: a retrospective population-based cohort study in Lithuania

**DOI:** 10.1192/j.eurpsy.2026.10658

**Published:** 2026-07-31

**Authors:** M. Drevinskaite, A. Germanavicius, G. Smailyte

**Affiliations:** 1 Department of Public Health, Faculty of Medicine; 2 Laboratory of Cancer Epidemiology, National Cancer Institute; 3 Clinic of Psychiatry, Faculty of Medicine, Vilnius, Lithuania

## Abstract

**Introduction:**

There is a scarcity of research analyzing the risk of cancer in people with schizophrenia based on the type of antipsychotic medication used, especially for those antipsychotics, which are prescribed to the small subset of patients with treatment-resistant schizophrenia.

**Objectives:**

The aim of this study was to analyze the risk of cancer in people with schizophrenia who used clozapine in Lithuania between 2013 and 2021.

**Methods:**

A retrospective cohort study design was used to evaluate the association between clozapine exposure and cancer risk. In this study, we used individual patient records from the Lithuanian Cancer registry and the National Health Insurance Fund (NHIF). In the NHIF database, we identified patients who were reported with schizophrenia (International Classification of Diseases (ICD)-10 code F20) from 2013 to 2021, the type of antipsychotic medications, and dates of prescription. Standardized incidence ratios (SIR) for cancers were calculated as the ratio of the number of observed cancer cases in the clozapine user cohort to the expected number of cancers in the general Lithuanian population.

**Results:**

The final cohort comprised 3473 patients with schizophrenia treated with clozapine and 91 cancer cases were observed. Overall cancer risk in schizophrenia patients who used clozapine was lower in males, but not in females, SIR 0.69 (95% CI 0.52 – 0.92). In our study we observed statistically significant results in prostate cancer, particularly in time-dependent manner, when clozapine was used for more than three years SIR 0.47 (95% CI 0.22 – 0.98). Additionally, for corpus uteri cancer, the risk was significantly increased when clozapine use exceeded three years SIR 2.42 (95% CI 1.21 – 4.83).

**Conclusions:**

Current evidence on the incidence of solid cancers among schizophrenia patients who use clozapine remains very limited. The results of our study highlight the importance of further prospective studies with adequate follow-up to clarify the potential cancer risks and guide clinical decision making.

**Disclosure of Interest:**

None Declared

